# A Cluster Randomized Controlled Trial to Reduce Childhood Diarrhea Using Hollow Fiber Water Filter and/or Hygiene–Sanitation Educational Interventions

**DOI:** 10.4269/ajtmh.13-0568

**Published:** 2014-07-02

**Authors:** Erik D. Lindquist, C. M. George, Jamie Perin, Karen J. Neiswender de Calani, W. Ray Norman, Thomas P. Davis, Henry Perry

**Affiliations:** Department of Biological Sciences, School of Science, Engineering and Health, Messiah College, Mechanicsburg, Pennsylvania; Department of International Health, Program in Global Disease Epidemiology and Control, Johns Hopkins Bloomberg School of Public Health, Baltimore, Maryland; Fundación contra el Hambre–Bolivia, Zona Sopocachi, La Paz, Bolivia; School of Science, Engineering and Health, Messiah College, Mechanicsburg, Pennsylvania; Food for the Hungry–USA, Phoenix, Arizona; Department of International Health, Health Systems Program, Johns Hopkins Bloomberg School of Public Health, Baltimore, Maryland

## Abstract

Safe domestic potable water supplies are urgently needed to reduce childhood diarrheal disease. In periurban neighborhoods in Cochabamba, Bolivia, we conducted a cluster randomized controlled trial to evaluate the efficacy of a household-level hollow fiber filter and/or behavior change communication (BCC) on water, sanitation, and hygiene (WASH) to reduce the diarrheal disease in children less than 5 years of age. In total, 952 households were followed for a period of 12 weeks post-distribution of the study interventions. Households using Sawyer PointONE filters had significantly less diarrheal disease compared with the control arm during the intervention period, which was shown by diarrheal prevalence ratios of 0.21 (95% confidence interval [95% CI] = 0.15–0.30) for the filter arm and 0.27 (95% CI = 0.22–0.34) for the filter and WASH BCC arm. A non-significant reduction in diarrhea prevalence was reported in the WASH BCC study arm households (0.71, 95% CI = 0.59–0.86).

## Introduction

The lack of sustainable access to safe water and sanitation services along with poor hygiene practices result in high mortality rates, impoverishment, and diminished opportunities for many people in low-income countries of the world.^1^,^2^ Although the provision of water, sanitation, and hygiene interventions (WASH) is complex and multifaceted, safe domestic water is important to effective WASH-related initiatives. The Millennium Development Goal (MDG) target of halving the global proportion of those people without sustainable access to safe water down to 12% has been met 5 years ahead of the 2015 goal.^3^ However, many of the world's poorest nations, notably those nations in sub-Saharan Africa, will still fall short of this goal. Today, the lack of access to safe water remains a serious concern for nearly 783 million persons, and by 2015, when these global objectives are supposed to be met, there will still be approximately 600 million persons without access.^3^

Diarrheal disease is the primary health threat that results from poor water quality. About 3.61% of the total disability-adjusted life year (DALY) global burden of disease is attributed to diarrhea, which is the cause of some 1.45 million deaths annually.^4^,^5^ Most of these deaths are among children under the age of 5 years, with diarrhea being the second largest cause of mortality in this age cohort worldwide.^6^

As of 2010, 71% of the rural population in Bolivia had access to improved drinking water sources (51% piped on premises and 20% other improved), which shows progress compared with 57% (33% piped on premises and 24% other improved) in 2000.^2^ However, statistics from 2004 in periurban Cochabamba (specifically in Districts 8 and 14) show that only 11.2% of households possessed piped infrastructure in 2004.^7^,^8^ In these districts, 71.8% of people receive water delivered by tanker trucks that are filled from artesian wells/cisterns located at the northern toe slope of the Cochabamba valley.^7^,^8^

Diarrheal surveillance conducted across Bolivia in 1998, 2003, and 2008 revealed that diarrhea prevalence for children under 5 years of age, the highest risk age group, has been on the rise: 19.2%, 22.4%, and 31.3%, respectively. The diarrhea prevalence in the Department of Cochabamba was 36.2% in 2008; this department includes the city of Cochabamba and the surrounding towns and communities. For the same period across Bolivia, there was little difference in diarrhea prevalence between households (with children under 5 years old) with and without improved municipal drinking water sources (31.0% and 32.5%, respectively).^9^

Safe domestic potable water supplies that are low in cost and easy to maintain are needed if a sustainable impact is to be made on childhood diarrheal disease in poor communities in low-income countries.^10^ Treatment of water against microbial contamination is vital to reducing morbidity in these communities. In areas where municipal sanitation and water supply infrastructure are lacking or in poor condition, household-level point-of-use (POU) water filtration can provide a safe, inexpensive solution.^11^ A wide variety of household filters are available on the market today, but few are low cost and easily maintained. Filters that use ultraviolet light or ozone are effective against microbial pathogens but require electricity, which makes them non-applicable or too costly in many settings. Several studies have examined the effectiveness of gravity-fed filters, particularly biosand and ceramic filters.^12^–^16^ However, long-term use of biosand filters has been met with limited success in transient communities because of high maintenance requirements. Although ceramic filters are effective, they can be cumbersome, difficult to clean, and susceptible to fracturing during distribution because of their fragility (Montes O, Fundación contra el Hambre-Bolivia, personal communication).^12^ Recently, the Sawyer Corporation and Messiah College partnered to design a gravity-fed biological filter system that uses a locally available receptacle that is easily maintained by household members.

**Figure 1.**
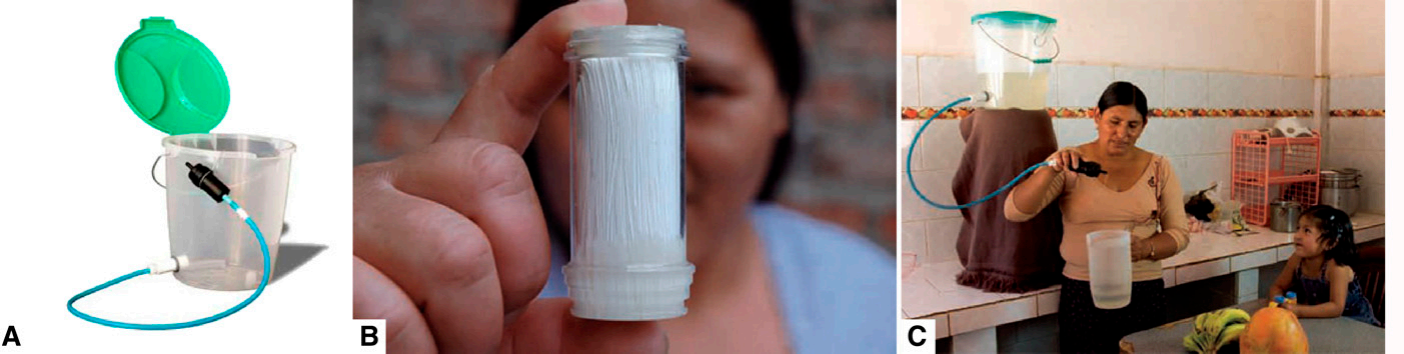
(**A**) The PointONE Filter produced by the Sawyer Corporation attached to a bucket with a lid was used in this study. (**B**) A transparent version of hollow fiber filter cartridge located within the filter casing. (**C**) A PointONE Filter bucket system in use and modeled for depiction in the filter training manuals used in the study.

The Sawyer PointONE (Sawyer Corporation, Safety Harbor, FL) is a POU filter that allows water to gravitationally flow into a 0.1-μm porous hollow fiber membrane bundle (Figure 1). This filter is attached to a hose that is coupled to a bucket in which unfiltered water is housed. An independent study testing three filter units in triplicate found that this filter system was successful in removing 5 log all protozoan parasites (*Giardia lamblia* and *Cryptosporidium parvum*) and 6 log bacteria (*Klebsiella terrigena*) tested in the laboratory.^17^ Filter flow rates reported by the Sawyer Corporation range from 32.8 to 99.2 L/hour depending on variables such as head pressure, altitude, and unit variability.^18^ Filter clogging can also affect flow rates. However, a backflow syringe that is provided with each unit makes cleaning easy and intuitive.

This paper presents findings from a cluster randomized controlled trial of two WASH interventions conducted in periurban, low-income communities on the periphery of Cochabamba, Bolivia. The primary objective was to evaluate the efficacy of using the PointONE Filter and/or disseminating WASH behavior change communication in significantly lowering the diarrhea prevalence among children under 5 years of age compared with a control group not receiving these interventions. The target cohort consisted of children under 5 years of age residing in the study catchment area. Adherence to the prescribed intervention was measured during the study period by using reported filter usage as a non-direct proxy of water treatment behaviors.

## Materials and Methods

### Setting.

The study population was located within eight economically depressed and ethnically marginalized (indigenous) periurban zones southeast and adjacent to the city of Cochabamba (located in the Cochabamba Department of Bolivia). Study sites were selected that lacked treated municipal water and sanitation through piped infrastructure (water mains and sewer lines). Because these periurban communities fall outside the zone of Cochabamba's municipal water supply, most households have water delivered by private providers using tanker trucks. These providers draw water from artesian wells located at the toe slope of the precordillera that flanks Cochabamba to the northeast. The present study was conducted in collaboration with the Fundación contra el Hambre–Bolivia (Food for the Hungry) in areas where they had existing community partnerships.

### Study design.

**Figure 2.**
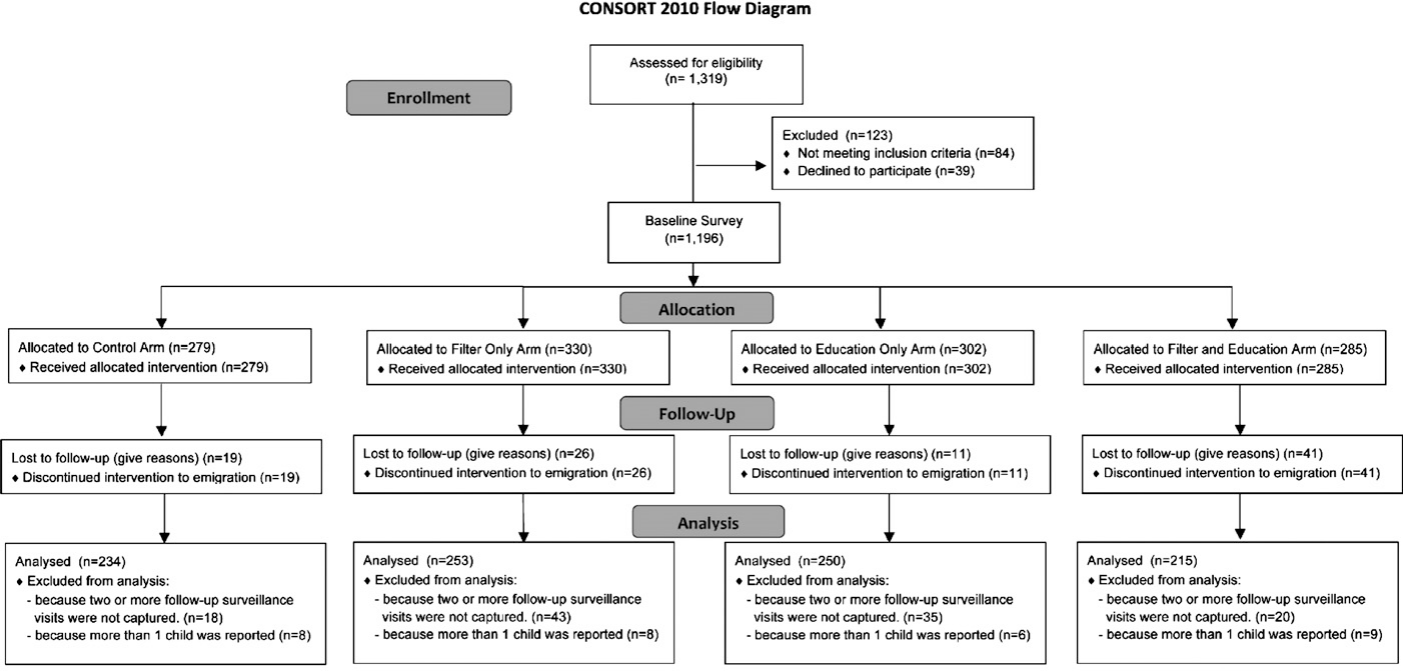
Intervention assignment and completed follow-up using CONSORT 2010 (CONsolidated Standards Of Reporting Trials).

The study design was a cluster randomized controlled trial with four study arms: (1) a control arm that received teachings on life skills (e.g., budget and family skills) not related to water and sanitation; (2) an arm that received a PointONE Filter and a 30-L bucket (with lid) with training on use and maintenance (filter arm); 3) an arm that received WASH behavior change communication (BCC), including basic water treatment training (i.e., boiling excluding filtration; WASH BCC arm); and (4) an arm that received a PointONE Filter and 30-L bucket (with lid) plus WASH education (filter and WASH BCC arm). Six to fifteen households with qualifying children were arranged into care groups, each with their own care group volunteer (CGV) that was trained to educate household representatives in their respective treatment/control arm lessons and collect monthly data during intervention surveillance. CGVs were recruited through Food for the Hungry. Sizes of individual care groups varied because of three factors: geographic proximity of participant households, local density of qualifying households, and availability of a CGV (leader) within locally grouped households. Between four and nine care groups were then arranged into a larger geographically based cluster (within the same neighborhood). Each study arm consisted of four treatment clusters, with a total of 16 treatment clusters for the study. By the beginning of the intervention phase, 1,196 households were assigned to the four study arms (Figure 2

). After geographically based treatment clusters were constructed, control and intervention types were randomly assigned until four of each type were achieved. There were four geographic zones. Each zone was divided into four neighborhoods that were randomized to the four treatment arms. The randomization was done at the neighborhood level. Treatment was assigned with a random number generator. Randomization was used to ensure that the populations were similar across study arms (Table 1).

### Power calculation.

Our power calculation was conducted in G*Power 3.1.3. Based on national statistics provided by Coa and Ochoa,^9^ we estimated that the diarrheal prevalence for the control area would be 35%.^19^ We anticipated that the difference in diarrhea prevalence attributed to the intervention in each of our study arms would be 20% based on the work by Clasen and others.^20^ To calculate a 20% difference in diarrhea prevalence between each of our intervention and control arms with a power of 80% and a type 1 error of 0.05, we calculated that we would need a sample size of 300 households in each our arms; therefore, our total sample size would need to be 1,200 households.^20^

### Eligibility and enrollment.

Between November and December of 2009, the aforementioned neighborhoods were canvassed by the study team in partnership with community leaders and directors to identify children who met our eligibility criteria. To be included, children needed to (1) be less than 60 months of age, (2) live in squatter or low-income rental housing, (3) receive their primary drinking/household water from a non-municipal source, and (4) live in a household that lacked access to a direct municipal sewer line. Enrollment was limited to one child per household, and signed consent was obtained for each household primary caregiver.

Each participating household's location was mapped using Google Earth v. 5.2 to facilitate study arm placement and follow-up and track long- or short-form survey designation.

### Interventions.

In this study, we examined POU water purification using a Sawyer PointONE hollow fiber filter and BCC on key WASH messages. The following study arms were used in the experimental design. (1) Filter: participants in this study arm received a Sawyer PointONE filter and a 30-L bucket (with lid) as well as weekly lessons by CGVs on the assembly, use (for drinking and cooking), cleaning, and long-term maintenance of the filter. If damage occurred to a system component during the study, then a replacement was provided. (2) WASH BCC: weekly WASH messages from CGVs on personal and family hygiene, sanitation, boiling and chlorine-based water treatments (excluding filtration), vitamin A, hygienic food preparation and cleaning, and parasite prevention were provided to this study arm. (3) Filter and WASH BCC: this study arm covered all equipment training and messaging used in the aforementioned filter and WASH BCC arms of the study. (4) Control: participants received weekly messages from CGVs on life skills and attitudes, such as household budgeting, value of children, responsibility to care for children, principles in family unity, and basic environmental stewardship. Given that drawing people into a social network may help behaviors to spread more extensively through that network, it was decided that the control arm should use a care group structure similar to the other intervention arms but not include promotion of water and sanitation behavior.^21^–^24^

In addition to the intervention-specific lessons given above, every study participant was taught basic lessons on diarrheal transmission (biological versus cultural beliefs-based), prevention and treatment, prevention of dehydration, and how to feed a sick child. At the close of the study, control and education intervention arm participants who completed the study term received filters, buckets, lids, and education on their use.

### Measurements.

Health technicians conducted a pre-intervention baseline Knowledge Practices and Coverage (KPC) survey of each household's primary caregiver between January 18 and March 3, 2010. This survey collected the following information: sociodemographic information, water source at home, and caregiver water treatment practices.

The intervention phase began for the three treatment arms of the study on March 15, 2010. From April to July of 2010, 2-week recall data on the presence of diarrhea and filter usage (in filter intervention arms) were gathered from each primary caregiver of a child enrolled in the study and reported to CGVs at the beginning of the second week of each month. All intervention arm households received their respective interventions (filters and/or WASH BCC) from CGVs by May of 2010. Therefore, our analysis is based on May to July of 2010.

Data forms for KPC and monthly intervention surveys were collected using Pocket PC Creations v. 5.0 for rapid and consistent data entry into handheld personal computers (HP iPAQ 110 Windows Mobile Handheld) and directly downloaded into a Microsoft Access 2007 project database.

### Fieldworkers and CGVs.

All study staff used in the present study were recruited by Fundación contra el Hambre–Bolivia (Food for the Hungry). Three health technicians, one monitoring and evaluation technician, and one field supervisor received training in adult educational methods, barrier analysis, use of Quality Improvement and Verification Checklists (QIVCs), interviewing techniques, and the Care Group Model.^25^,^26^ Staff also received periodic refresher training on these topics.

CGVs were originally selected during meetings held in the community. Groups of constituent caregivers met to select their CGV. Participation as a CGV was voluntary, and the CGV was free to resign at any time during the study. During the meetings, staff explained the roles and responsibilities involved and then assisted community members in voting for their CGV. The following criteria were used in CGV selection: female, a third-grade education minimum, ability to read and write, had one child participating in the study, and had an interest in learning about health. If a CGV resigned, study staff gathered the group together to select a replacement or asked another caregiver in the care group to take on the CGV leader role.

All CGVs received a 12-session training module by trained study health technicians. Health technicians taught CGVs how to present the educational modules, which included peer learning sessions, where health promotion materials were presented to other CGVs for practice. Health technicians took this opportunity to observe the health promotion skills of the CGVs and provide them with feedback. For each study arm, CGVs were given a session outline and educational materials in the form of flip charts. Each CGV taught using these materials during her biweekly home visits or care group meetings.

### Data analysis.

Our study objective was to determine if the filter and WASH BCC interventions were effective in significantly lowering the diarrhea prevalence in our study population compared with the control group. The main outcome in the present study is the percentage of surveillance visits for each child where a caregiver reported a child having a diarrhea episode in the past 2 weeks. This percentage was calculated by dividing the number of surveillance visits when diarrhea was reported by the total number of surveillance visits for each child. For this analysis, only surveillance visits in May, June, and July of 2010 were included, because not all study households received the intervention in the first surveillance visit in April of 2010. We also calculated the monthly diarrhea prevalence for each study arm by dividing the number of surveillance visits when diarrhea was reported by the total number of surveillance visits for each study arm. To determine the diarrhea prevalence ratios for each of the three intervention arms compared with the control group, we used generalized estimating equations (GEEs) with a Poisson regression to account for clustering within study geographic clusters.^27^ Although we measure diarrhea prevalence over time, we summarized diarrhea for each child over the study period before analysis. We use GEEs to approximate the prevalence ratios between study arms, which are close but not exactly the same as from the raw data, because GEEs account for the geographic clustering between children. We do not rely on the estimates of variability from GEEs, because the number of clusters is too small. Two-sided Wilcoxon rank sums using the exact method were calculated to determine if there were significant differences between the three study intervention arms and the control arms. GEEs could not be used to determine these *P* values because of the low variance estimate attributable to the small number of study clusters. These variance estimates can be too low when the number of geographic clusters is not large, and they are not reliable enough for *P* values to be calculated.^28^ All analyses were performed using SAS, version 9.3 (SAS Institute Inc., Cary, NC).

### Filter usage.

Filter usage data were gathered for each constituent household for each month of the 4 intervention months. Household caregivers reported filter usage estimates to CGVs with the following categories: never, seldom, sometimes, almost always, or always.

### Focus group.

Household primary caregivers (*N* = 40) living in the Uspha Uspha community provided researchers with opinions about the PointONE filter in a focus group study in June of 2010.

### Ethics.

The study protocol was approved by the Messiah College Institutional Review Board. Signed informed consent forms were obtained from all study respondents. All household primary caregivers that wanted to end their participation in the study were allowed to do so at any time, and they were provided an opportunity to decline participation at the beginning of every CGV or study personnel visit. All requisite permissions were obtained from governmental authorities before the enrollment phase of the study commenced.

## Results

The study cohort consisted of a total of 1,196 households with at least one child residing in the home that was less than 5 years of age. Fifty-three percent of the respondents were female, and the median age was 20 months (range = 4–40 months). A CONSORT 2010 diagram of study enrollment and participation is provided in Figure 2 (CONsolidated Standards Of Reporting Trials). Of 1,196 households that began the intervention phase of the study, 97 households were lost because of emigration. In total, 195 households were excluded from the final analysis: 164 households were excluded, because they were missing two or more household visits between May and July of 2010, and 31 households were excluded, because more than one child was enrolled in the household. Selected pre-intervention baseline KPC characteristics for all households profiled by the respective study arm are presented in Table 1. Fifty-three percent of households in our intensive KPC survey reported no sanitation option in their home. Flush toilet connected to a septic tank was reported in 25% of households, pit latrine without a slab was reported in at 13% of households, and pit latrine with a slab was reported in at 9% of households. There were no significant differences observed across study arms. Likewise, the location of toilets and latrines ranged from 11% inside or attached to the dwelling and 77% detached from the dwelling but inside the dwelling property to 11% outside of the dwelling property and < 1% indicated as other. Demographic features of the study participants, such as caregiver and child ages and sex ratios, were similar across control and filter intervention groups, and they are profiled in Table 1. The loss of 97 (10.7%) participants to emigration in 4 months reflects the transient nature of the study communities. The reason for the slightly higher loss to follow-up in the filter and BCC arm is unknown.

**Figure 3.**
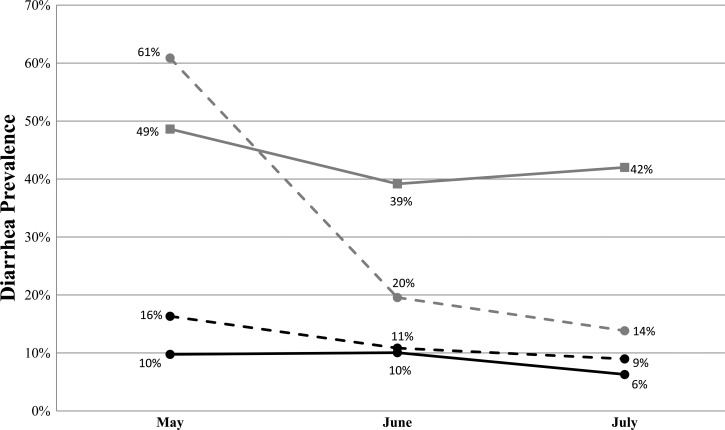
Diarrhea prevalence by study month. Percent prevalence for each study arm is shown above each point. Dashed black line = filter and WASH BCC arm; solid black = filter arm; dashed gray = WASH BCC arm; solid gray = control arm.

Diarrheal disease prevalence and stratified diarrhea prevalence ratios are specified in Figure 3

and Table 2 and were based on CGV reported monthly reported data. The diarrhea prevalence ratio (DPR) effect estimate compared with the control group for the filter arm was 0.15 (95% confidence interval [95% CI] = 0.10–0.22) or a mean reduction in diarrheal disease of 85% after controlling for clustering within geographic clusters. Additionally, the filter and WASH BCC arm DPR effect estimate was 0.22 (95% CI = 0.16–0.30) or a 78% mean reduction in diarrheal disease. The lower mean reductions in diarrhea prevalence were significant for both the filter and filter and WASH BCC study arm households compared with the control arm households; both had identical *P* values of 0.0286 using the Wilcoxon rank sums with the exact method.

### Filter usage.

We defined a filter user as someone who reported using the filter always or almost always in a given intervention month. Of participant households in the filter arm, 97% reported being filter users, and 90% reported being filter users in the WASH BCC and filter arm. The reason for the higher filter usage in the filter arm compared with the WASH BCC and filter arm is unknown.

### Focus group.

Commonly stated positive feedback regarding the water filter included ease of use, clearer appearance of water, and better taste and smell than their source water. Many stated that they believed that the filter was more effective and advantageous in purifying water than traditionally boiled or chemically treated methods. Participants made important design recommendations, such as having a cleaner-looking filter hose, a tethered filter spout cap, and a stronger filter storage hook.

## Discussion

This study represents the first cluster-based randomized controlled trial on the use of household-level hollow fiber POU water filters (Sawyer PointONE) in the field. Results from this factorial design study show statistically significant reductions in diarrheal disease among children less than 5 years of age in filter and filter and WASH BCC study arm households compared with control households. Although a reduction in diarrhea prevalence was observed in the WASH BCC arm households, differences were not statistically significant. Likewise, no additional reductions in diarrheal disease were observed in filter and WASH BCC households compared with the households that only had the filter.

Piped treated municipal water delivery is still an important developmental goal for low-income populations. However, the Sawyer PointONE POU filter seems to be an effective interim tool for use in communities using microbial-contaminated water sources. Compared with ceramic candle filters (i.e., Katadyn 2110070), the PointONE filter costs less: $216 for the ceramic filter compared with $60 for the PointONE filter. The PointONE filter also has the added benefit of being easy to transport and install, making it a good option in combating waterborne disease in emergency settings. In addition, the PointONE filter has a manufacturer-specified 10-year minimum lifespan. Filter usage has an inherent environmental benefit over boiling water, which places high demand on collecting firewood and in turn, degrades puna, forests, and scrubland habitat, contributing to habitat loss pressures for endemic birds and native plants.^29^

In the present intervention, we did not observe a significant impact of the WASH BCC study arm. The findings suggest that the distribution of the filter alone was sufficient to reduce diarrhea outcomes. This finding is consistent with metaregressions by Brown and others,^30^ Stauber and others,^31^ and Hunter^32^ on filter interventions conducted in Asia using ceramic water purifiers (CWPs) and biosand filters (BSFs).^30^–^32^ Results from this study suggest that hollow fiber filters can reduce diarrhea prevalence in children under the age of 5 years an additional 6–13% compared with 72% reported in a study on CWPs in Bolivia.^14^ Compared with the DPR values reported in a study in Cambodia on CWPs (0.58, 95% CI = 0.41–0.82) and iron-rich CWPs (0.65, 95% CI = 0.46–0.93), the diarrheal disease prevalence reductions associated with the PointONE filter seem to be noteworthy.^30^ Likewise, in another Cambodian study on BSF interventions, a 22% reduction in prevalence of diarrhea was observed, still markedly lower than the effect sizes reported with the PointONE filter.^31^ Lastly, in a meta-analysis on 33 reports from 21 different countries conducted by Clasen and others,^21^ chlorination and flocculation disinfection studies estimating the intervention effect in children under 5 years of age using longitudinal prevalence ratios showed that they were less effective (0.91, 95% CI = 0.82–1.02 and 0.42, 95% CI = 0.13–1.37, respectively). Intervention expenses vary depending on the technologies and level of human resources required, and therefore, it is important for healthcare and social work organizations to weigh the costs and benefits of using the filter alone or with educational modules.

There are several limitations to the present study. One limitation is the short duration of the study intervention period of 3 months. A future study should be conducted for a longer period of time to assess the sustainability of this filter technology over time. A second limitation is the small amount of geographic clusters in the present study. If the number of clusters had been larger, we would have been able to use GEEs to detect significant differences between our study arms and adjust for study covariates, such as sociodemographic characteristics in the study population. A third study limitation is that the same CGVs that administered the study interventions collected diarrheal surveillance data, which could result in differential reporting bias, potentially leading to underreporting of diarrhea in the intervention versus the control arms. A fourth study limitation is that we used a 2-week recall on the presence of diarrhea and did not collection information on the number of episodes or the severity or duration of episodes. It has been established that recall periods beyond 2 days can underreport events.^33^–^36^ Because of this tendency, we measured diarrheal disease prevalence conservatively, and therefore, the effect sizes given in this study are likely underestimates. Lastly, an important design limitation is the lack of study blinding with the implementation of a placebo/sham filter study arm. Members of the research team and the local administration of Food for the Hungry–Bolivia voiced many logical and ethical reservations to this aspect of the study design.^37^ Therefore, there exists a potential for participant-level reporting bias in the intervention study arms.

## Conclusion

The findings from the present study suggest that the Sawyer PointONE filter can be an effective tool to reduce diarrheal prevalence in children under the age of 5 years. Additional research is needed to evaluate this filter in different settings globally. A longer longitudinal study would also be helpful in providing a stronger evidential base for the sustainability of the interventions' efficacy. Also, implementation in diverse environmental settings (such as areas with disparate rainfall regimes) and diverse cultural settings (such as areas with different water, sanitation, and hygiene practices) would be helpful. Likewise, a comparative rapid assessment of water-borne disease prevalence in disaster relief populations with groups using Sawyer PointONE hollow fiber filters, other POU filters (e.g., ceramic candles), and chemical treatment (e.g., Aquatab and chlorine-based treatments) could be of additional use.

## Figures and Tables

**Table 1:** Population characteristics by study arm

	Control (*N* = 220)	Education (*N* = 246)	Filter (*N* = 235)	Filter and education (*N* = 203)
Age of child, months ± SD (range)	20 ± 8.9 (4–38)	21 ± 9.0 (2–38)	20 ± 9.0 (3–40)	19 ± 8.7 (3–38)
Percent female, %	45	50	51	45
Primary language spoken by household, %				
Aymara	3	2	4	4
Spanish	59	44	59	36
Quecha	38	54	37	60
Caregiver years of education, %				
None	7	12	10	18
1–5	24	35	21	21
5–10	33	31	32	34
Greater than 10	36	22	37	26
Floor type in household, %				
Concrete	84	77	84	77
Title	14	5	11	8
Brick	0	1	0	3
Dirt	2	17	5	10
Other				1
Main source of drinking water, %				
Rain water collection	< 1	< 1	0	0
Water coolers	12	6	7	6
Tanker truck	83	92	84	91
No water given	< 1	< 1	< 1	1
Piped water in the dwelling	3	0	6	0
Piped water outside of dwelling	< 1	< 1	2	0
Public tap	0	0	< 1	0
Dug well	0	0	< 1	< 1
Surface water	0	0	0	< 1
Other	< 1	< 1	< 1	1
Reported water treatment, %				
Boil	71	61	70	64
Use of bleach or chlorine	0	0	1	0
Use of commercial water treatment product	3	< 1	0	1
Ceramic filter, sand filter, or biofilter	0	0	0	0
Solar disinfection	3	< 1	2	2
Straining through cloth	0	< 1	< 1	< 1
Sedimentation of water (allowing to stand before drinking)	0	0	0	< 1
Other	0	0	0	< 1
Loss to follow-up, %	7	8	4	14

**Table 2:** Diarrheal disease prevalence and intervention effect estimates

Study arm	May 2010 diarrhea period prevalence (%)	June 2010 diarrhea period prevalence (%)	July 2010 diarrhea period prevalence (%)	Diarrhea prevalence over 12-week period (%)	Diarrhea prevalence ratio (95% CI)*	*P* value†
Control (*N* = 220)	49	39	42	43	−	−
WASH BCC (*N* = 246)	61	20	14	30	0.71 (0.59–0.86)	0.0857
Filter (*N* = 235)	10	10	6	9	0.21 (0.15–0.30)	0.0286
Filter and WASH BCC (*N* = 203)	16	11	9	12	0.27 (0.22–0.34)	0.0286

* Calculated using a GEE using a Poisson distribution adjusted for study clusters.

† *P* values were calculated using Wilcoxon scores (rank sums) with the exact method (two sided).
